# Efficacy and mechanism of intermittent fasting in metabolic associated fatty liver disease based on ultraperformance liquid chromatography-tandem mass spectrometry

**DOI:** 10.3389/fnut.2022.838091

**Published:** 2022-11-14

**Authors:** Jiang Deng, Dandan Feng, Xiaoli Jia, Song Zhai, Yixin Liu, Ning Gao, Xin Zhang, Mei Li, Mengnan Lu, Chenrui Liu, Shuangsuo Dang, Juanjuan Shi

**Affiliations:** ^1^Department of Infectious Disease, The Second Affiliated Hospital of Xi'an Jiaotong University, Xi'an, China; ^2^Xi'an Jiaotong University Health Science Center, Xi'an, China

**Keywords:** metabolic associated fatty liver disease (MAFLD), intermittent fasting (IF), time-restricted feeding (TRF), lipidomics, triglycerides

## Abstract

**Objectives:**

Drug treatment of metabolic associated fatty liver disease (MAFLD) remains lacking. This study analyzes the efficacy and mechanism underlying intermittent fasting combined with lipidomics.

**Methods:**

Thirty-two male rats were randomly divided into three groups: Normal group, administered a standard diet; MAFLD group, administered a 60% high-fat diet; time-restricted feeding (TRF) group, administered a 60% high-fat diet. Eating was allowed for 6 h per day (16:00–22:00). After 15 weeks, liver lipidomics and other indicators were compared.

**Results:**

A total of 1,062 metabolites were detected. Compared with the Normal group, the weight, body fat ratio, aspartate aminotransferase, total cholesterol, low-density cholesterol, fasting blood glucose, uric acid, and levels of 317 lipids including triglycerides (TG) (17:0_−_18:1_−_20:4) were upregulated, whereas the levels of 265 lipids including phosphatidyl ethanolamine (PE) (17:0_−_20:5) were downregulated in the MAFLD group (*P* < 0.05). Compared with the MAFLD group, the weight, body fat ratio, daily food intake, and levels of 253 lipids including TG (17:0_−_18:1_−_22:5) were lower in the TRF group. Furthermore, the levels of 82 lipids including phosphatidylcholine (PC) (20:4_−_22:6) were upregulated in the TRF group (*P* < 0.05), while serum TG level was increased; however, the increase was not significant (*P* > 0.05). Enrichment analysis of differential metabolites showed that the pathways associated with the observed changes mainly included metabolic pathways, regulation of lipolysis in adipocytes, and fat digestion and absorption, while reverse-transcription polymerase chain reaction showed that TRF improved the abnormal expression of *FAS* and *PPAR*α genes in the MAFLD group (*P* < 0.05).

**Conclusion:**

Our results suggest that 6 h of TRF can improve MAFLD *via* reducing food intake by 13% and improving the expression of genes in the PPARα/FAS pathway, thereby providing insights into the prevention and treatment of MAFLD.

## Introduction

Non-alcoholic fatty liver disease (NAFLD), also known as metabolic associated fatty liver disease (MAFLD), has become the most prevalent liver disease worldwide. In 2016, a meta-analysis of 86 studies in 22 countries showed that the global prevalence of NAFLD was 25.2%, with Africa having the lowest prevalence at 13.5%, and the Middle East having the highest prevalence at 31.8%, followed by Asia at 27.4% (1–3). The prevalence of NAFLD in China has been reported at 29.2% (4). Although the risk of MAFLD increases with age (5), especially in women, an increasing number of cases of MAFLD have been reported in children. To date, no specific drug therapy has been established, and currently available treatments primarily focus on diet control and physical activity (6).

Intermittent fasting (IF) is an ancient dietary therapy characterized by zero or very low-calorie intake (usually for more than 12 h) alternated with normal eating to prevent or treat diseases (7). IF, which includes time-restricted feeding (TRF), alternate-day fasting, and 5:2 modified fasting, can improve metabolic syndrome, prolong life expectancy, and improve cognition (8, 9). However, data on the effectiveness of IF in improving MAFLD are limited, while controversy remains concerning whether IF aggravates MAFLD due to excessive fat breakdown.

Therefore, this study analyzes the food intake, therapeutic effect, and underlying mechanism in rats with MAFLD undergoing 6 h TRF to provide novel insights into the treatment of MAFLD.

## Materials and methods

### Animals and study protocol

Thirty-two specific-pathogen-free male Sprague-Dawley (SD) rats with a body weight of 185–245 g were purchased and raised in the Animal Center of Xi'an Jiaotong University [SYXK (Shaanxi) 2020-005]. After 1 week of adaptive feeding, 10 rats were randomly selected in the Normal group (262.4 ± 12.5 g) using the random number table method and fed a normal diet (10 kcal% fat); 12 rats were assigned to the MAFLD group (263.9 ± 20.1 g) and had unrestricted access to a high-fat diet (60 kcal% fat); 10 rats were assigned to the TRF group (264.7 ± 19.5 g), in which access to a high-fat diet (60 kcal% fat) was restricted from 16:00 to 22:00 daily. No significant difference in baseline body weight between the three groups was observed (*P* = 0.958). The high-fat diet was purchased from Changzhou SYSE BIO (China); the carbohydrate, protein, and fat energy accounted for 20, 20, and 60%, respectively. All rats had access to water *ad libitum* and were exposed to a 12:12 day:night cycle at a constant temperature of ~22°C. During the 15-week experimental period, food intake was measured daily, while general conditions such as skin color, hair, and animal behavior were observed. The rats were weighed every week.

This study was approved by the Biomedical Ethics Committee, School of Medicine, Xi'an Jiaotong University (2021-763).

### Biochemical parameters

At the end of week 15 and after overnight fasting for 12 h, the rats were anesthetized with an intraperitoneal injection of 1% pentobarbital sodium (40 mg/kg). Approximately 4 mL of blood was collected from the heart, incubated at room temperature for 1 h, and centrifuged at 3,500 rpm for 10 min to separate the serum. Fasting blood glucose (FBG), total cholesterol (TC), triglycerides (TG), low-density cholesterol (LDL), alanine aminotransferase (ALT), aspartate aminotransferase (AST), and uric acid (UA) were measured using an automatic biochemical analyzer (AU5811, Beckman Coulter, CA, USA). Body fat ratio was defined as = (epididymis fat + perirenal fat) / weight × 100%.

### Histopathology

Hematoxylin-eosin (HE) staining (10, 11): Perirenal adipose and liver tissue samples from each group, ~5 mm in size, were fixed in 4% paraformaldehyde, dehydrated, embedded in paraffin, sliced, stained with HE, and observed under a microscope as previously described (12).

Oil red staining: Liver tissue samples from each group, ~5 mm in size, were fixed in 4% paraformaldehyde, dehydrated, embedded in optimal cutting temperature compound, sliced, stained, sealed with glycerin gelatin tablets, and observed under a microscope. Four images were randomly selected from each section, and the tissue area occupied by lipid droplets, representing the fat content of the liver, was calculated using Image J software (v1.8.0, National Institutes of Health, USA).

### Liver lipidomics

Using ultraperformance liquid chromatography and tandem mass spectrometry (UPLC-MS/MS) with Metware Database and multiple reaction monitoring (MRM), the lipid metabolites in all samples were qualitatively and quantitatively assessed. Quality control (QC) samples were prepared from a mixture of sample extracts. During instrumental analysis, one QC sample for every 10 test samples was inserted to ensure the repeatability of the analysis process. The total ion chromatograms (TICs) of different QC samples were overlapped and analyzed (13–15).

Principal component analysis (PCA) and orthogonal partial least squares discriminant analysis (OPLS-DA) were combined to identify differential metabolites. Metabolites with fold change (FC) of ≥ 2 or ≤ 0.5 and variable importance in the projection of ≥ 1 were selected as differential metabolites. Kyoto Encyclopedia of Genes and Genomes (KEGG) database was used to annotate and display the differential metabolites and analyze the related metabolic pathways (15).

### Reverse-transcription polymerase chain reaction of liver

RNA was extracted using Total RNA Extraction Kit I (OMEGA, R6834-01). RNA concentrations were determined with spectrophotometric trace (NanoDrop, Thermo Fisher Scientific, Waltham, MA, USA). Total RNA was transcribed into cDNA (volume: 20 μL) following the manufacturer's instructions of PrimeScript RT Master Mix (TaKaRa, RR036A) and TB Green Premix Ex Taq II (TaKaRa, RR820A). We used ABI StepOne Plus (USA) to determine the relative abundance of the mRNAs of interest. All procedures were strictly conducted in accordance with the instructions. The expression of each gene was quantified using the 2^−Δ*ΔCt*^ method (11, 16).

The primer sequences used were peroxisome proliferator-activated receptor α (*PPAR*α) upstream primer (5‘-3‘) TCTGAACATTGGCGTTCGCAG and downstream primer CTCGTGTGCCCTCCCTCAAG; fatty acid synthase (*FAS*) upstream primer (5‘-3‘) AATTTGCTCGGCAGCACAAG and downstream primer GTCGCAGCGGTTAGCTTTTC; sterol regulatory element binding proteins-1c (*SREBP-1c*) upstream primer (5‘-3‘) GCCATGGATTGCACATTTGAAGA and downstream primer TGTGTCTCCTGTCTCACCCC; glyceraldehyde phosphate dehydrogenase (GAPDH) upstream primer (5‘-3‘) TACCCACGGCAAGTTCAACG and downstream primer CACCAGCATCACCCCATTTG.

### Statistical analysis

Normally distributed data were expressed as mean ± standard deviation, and one-way analysis of variance was used, while Fisher's least significant difference *t*-test or Tamhane's *T*^2^-test to determine significance. Skewed data were described *via* quartile spacing and compared using non-parametric and median tests. All rats were tested for routine parameters, such as body weight, body fat percentage, and serological indicators. No less than four biological replicates were considered for lipidomics and PCR.

Lipid data were analyzed using Analyst 1.6.1 software. The IBM SPSS 23.0 (IBM Corp., Armonk, NY, USA) was used for all statistical analyses. GraphPad Prism 8.0 software (San Diego, CA, USA) was used to generate graphs. Statistical significance was set at *P* < 0.05.

## Results

### Physical characteristics

**Figure 1:**
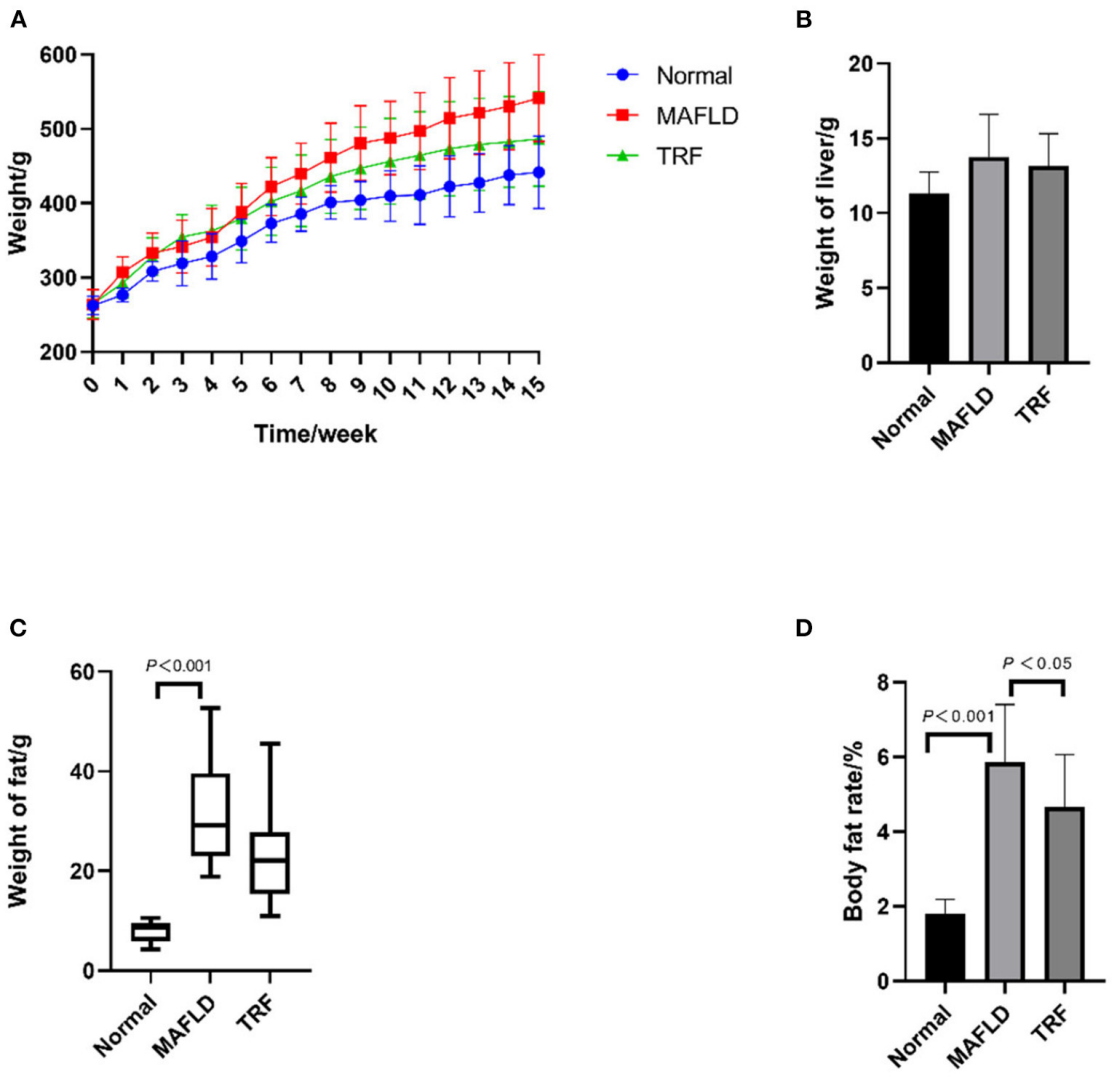
Physical characteristics. **(A)** Body weight from 1 to 15 weeks (g); **(B)** weight of liver (g); **(C)** weight of perirenal and epididymal fat (g); **(D)** body fat rate (%). The data are presented as means ± SD or percentile. Normal, rat fed a normal diet *ad libitum*; MAFLD (metabolic associated fatty liver disease), rat fed a high-fat diet *ad libitum*; TRF (time-restricted feeding), fed high-fat diet (60 kcal% fat) strictly only between 16:00 and 22:00 every day.

As shown in Figure 1A, at the end of week 15, the body weight difference between the three groups was significant (*P* = 0.005), with rats in both the Normal group (*P* = 0.001) and 6 h TRF group (*P* = 0.044) weighing less than those in the MAFLD group. As shown in Figure 1B, there was no significant difference in liver weight among the three groups. As shown in Figure 1C, the fat weight of the MAFLD group was significantly higher than that of the Normal group.

As shown in Figure 1D, the body fat ratio of the rats in the three groups was significantly different (*P* < 0.001); indeed, the body fat ratio of the rats in the MAFLD group was significantly higher than that of the rats in the Normal group (*P* < 0.001) and TRF group (*P* < 0.05), suggesting that TRF reduced body fat.

### Feeding behavior

**Figure 2:**
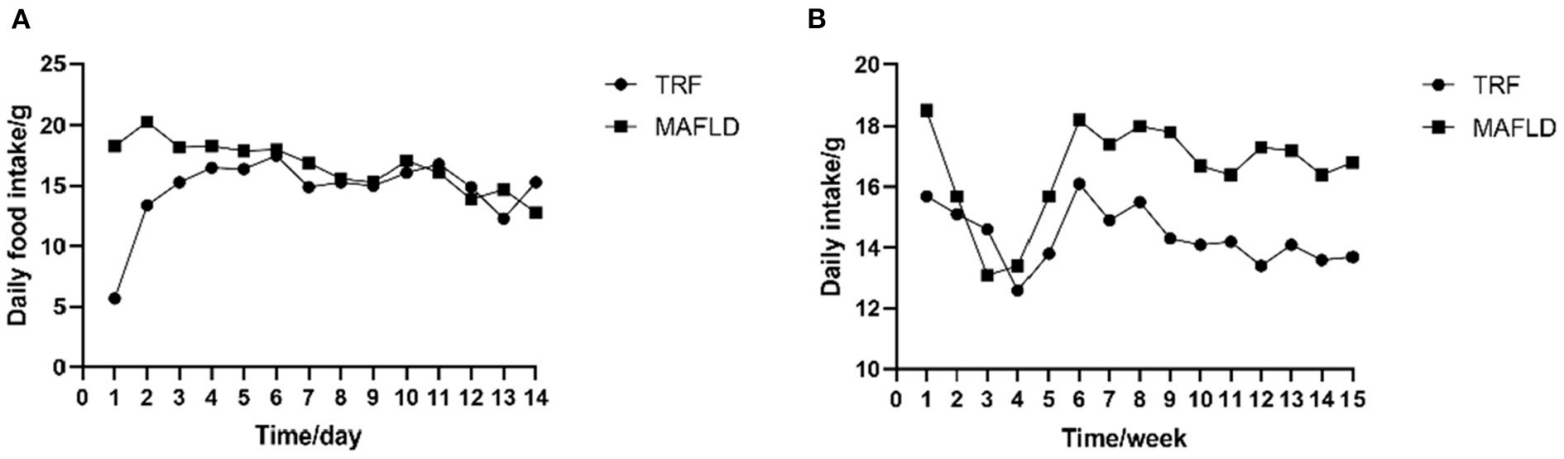
Feeding behavior. **(A)** Daily food intake of each rat from day 1 to 14; **(B)** average daily food intake of each rat from 1 to 15 weeks (g). MAFLD (metabolic associated fatty liver disease), rat fed a high-fat diet *ad libitum*; TRF (time-restricted feeding), fed high-fat diet (60 kcal% fat) strictly only between 16:00 and 22:00 every day.

The amount of food eaten was determined by subtracting amount of food administered from that remaining at the end of the eating period. As shown in Figure 2A, the food intake of rats in the 6 h TRF group was relatively low on day 1 after IF initiation; however, it increased significantly on day 2, as rats began to adapt to the fasting mode.

In the first 4 weeks of the study, the average daily food intake of both the MAFLD and 6 h TRF groups decreased, possibly due to rats not fully adapting to the high-fat diet, and subsequently increased on weeks 5–6 and remained relatively stable thereafter (Figure 2B).

During the entire experimental period, the average daily food intake in the 6 h TRF and MAFLD groups differed (*P* < 0.001), being 16.6 ± 1.6 g in the MAFLD group and 14.4 ± 0.9 g in the 6 h TRF group; ~13% of total calories were restricted.

### Biochemical parameters

**Figure 3:**
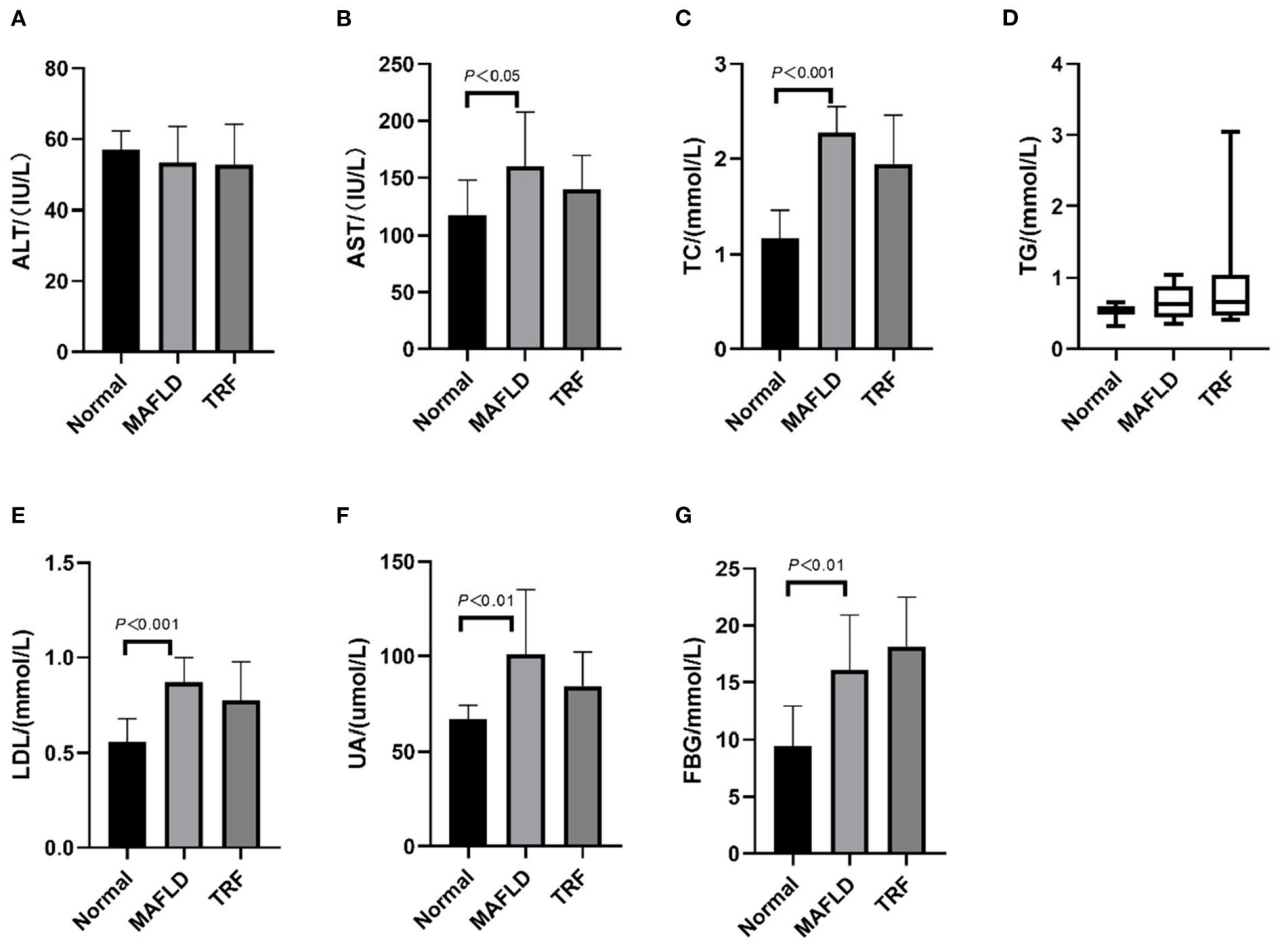
Serum indexes. **(A)** Alanine transaminase; **(B)** aspartate aminotransferase; **(C)** total cholesterol; **(D)** triglycerides; **(E)** low-density lipoprotein; **(F)** uric acid; **(G)** fasting blood glucose. The data are presented as means ± SD or percentile. Normal, rat fed a normal diet *ad libitum*; MAFLD (metabolic associated fatty liver disease), rat fed a high-fat diet *ad libitum*; TRF (time-restricted feeding), fed high-fat diet (60 kcal% fat) strictly only between 16:00 and 22:00 every day.

As shown in Figure 3, after 15 weeks of a high-fat diet, AST, TC, LDL, UA, and FBG levels in the MAFLD group were significantly higher than those in the Normal group (*P* < 0.05), whereas in the 6 h TRF group, AST, TC, LDL, and UA levels were reduced. However, the difference was not significant (*P* > 0.05).

Compared with that in the MAFLD group, the serum TG level in the 6 h TRF group was increased, although not significantly (*P* > 0.05).

### Histopathology

**Figure 4:**
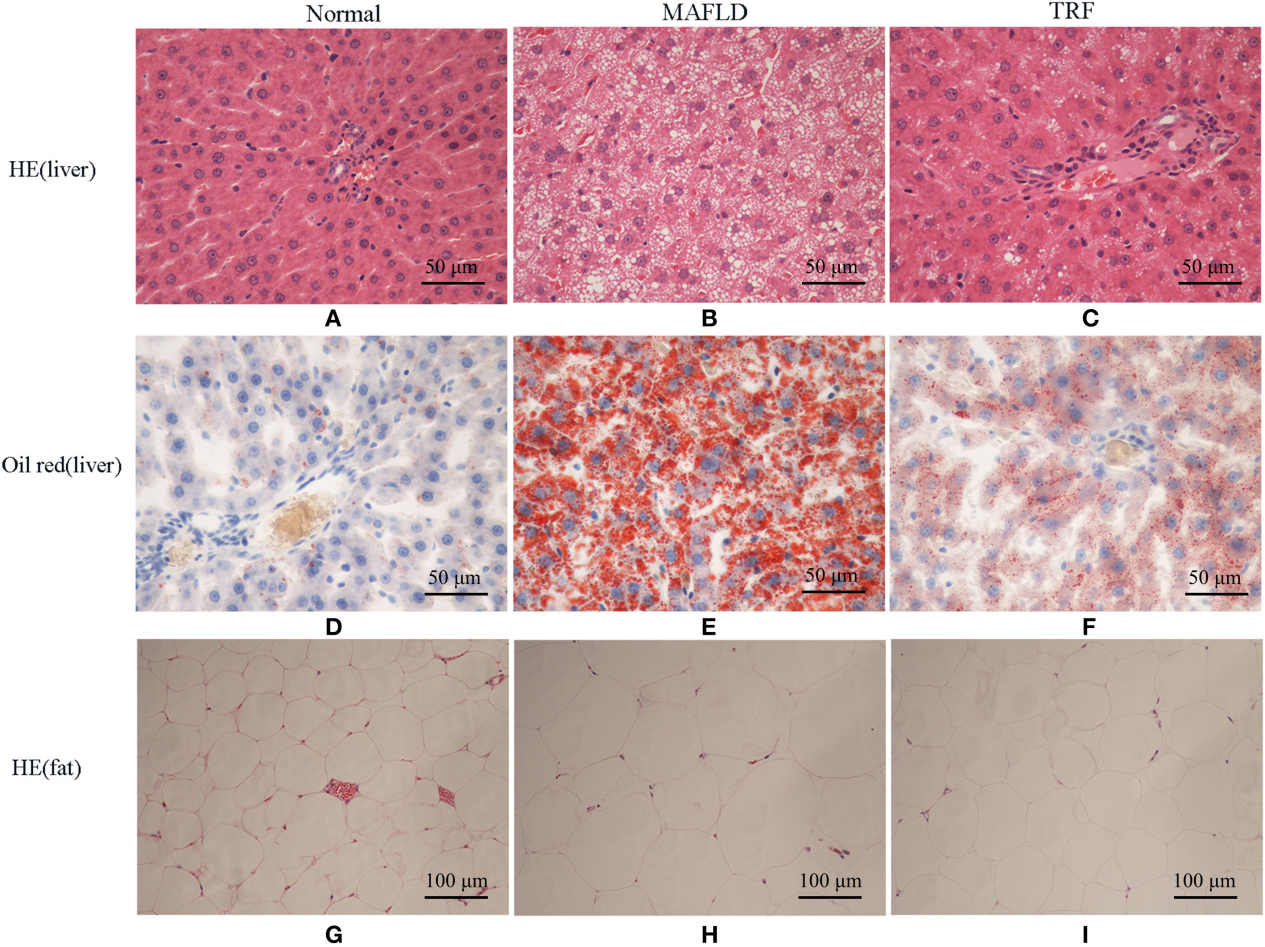
Pathological results of Normal group, MAFLD group and TRF group. **(A–C)** HE staining, **(D–F)** oil red staining of liver tissue (×400), and **(G–I)** HE staining of perirenal fat (×200). Normal, rats fed a Normal diet *ad libitum*; MAFLD (metabolic associated fatty liver disease), rats fed a high-fat diet *ad libitum*; TRF (time-restricted feeding), rats fed high-fat diet (60 kcal% Fat) strictly between 16:00 to 22:00 every day.

The Normal group showed normal hepatic cord structure and radially arranged hepatocyte morphology (Figure 4A). In the MAFLD group, the hepatic cord structure was disorganized, while the hepatic cells were significantly swollen. In most hepatic cells, vacuoles of varying sizes and numbers of lipid droplets were observed, while some cells showed obvious nuclear deviation (Figure 4B). The number of fat vacuoles decreased in the 6 h TRF group (Figure 4C).

**Figure 5:**
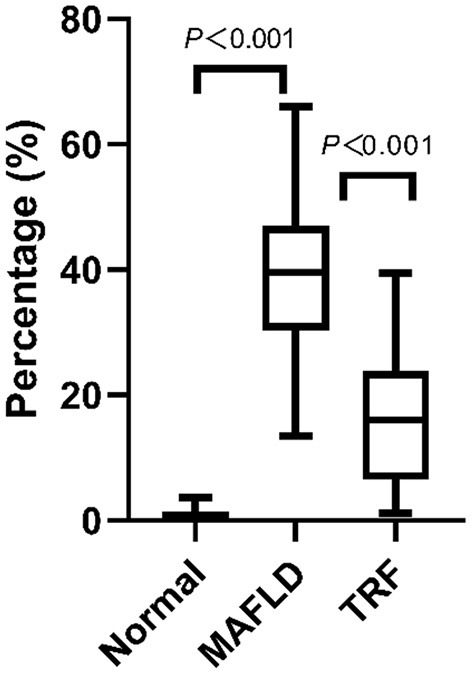
Area of lipid droplets in liver (%). Four images were randomly selected from each section, and the tissue area occupied by lipid droplets, representing the fat content of the liver, was calculated using Image J software (v1.8.0,National Institutes of Health, USA). The data are presented as percentile.

Oil red staining showed that the liver cells of the rats in the Normal group were slightly stained with scattered, red-stained lipid droplets (Figure 4D), while in the MAFLD group, several bulla-like red lipid droplets were observed in the cytoplasm (Figure 4E); liver lipids were significantly less deposited in the 6 h TRF group (Figure 4F). Compared with the Normal group, the MAFLD group had significantly higher fat content (*P* < 0.001), whereas the 6 h TRF group had significantly lower fat content in the liver than the MAFLD group (*P* < 0.001; Figure 5).

The adipose cells of rats were arranged regularly and uniformly (Figure 4G), whereas in the MAFLD group, cells showed different sizes, and were disordered (Figure 4H). The adipose cells in the 6 h TRF group were arranged more neatly than those in the MAFLD group with uniform cell size (Figure 4I).

### Liver lipidomics

**Figure 6:**
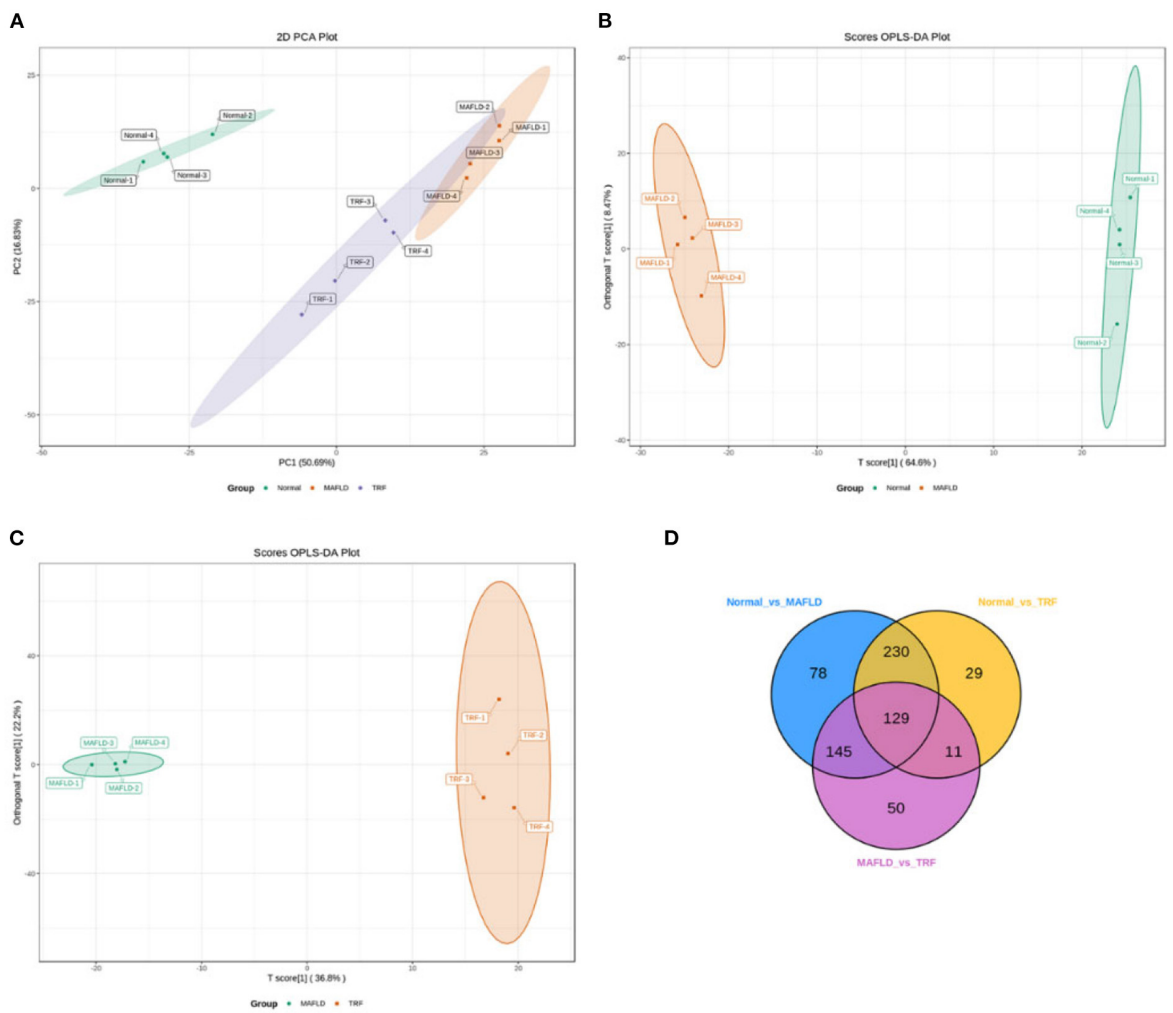
Liver lipidomics results. PCA for all groups **(A)**. OPLS-DA scores plots **(B,C)** of Normal vs. MAFLD and MAFLD vs. TRF, respectively. In the Venn diagram **(D)**, each circle represents a comparison group, the number of circles and overlapped parts represent the number of common differential metabolites between the comparison groups, and the number of non-overlapped parts represents the number of unique differential metabolites in the comparison groups. Normal, rats fed a normal diet *ad libitum*; MAFLD (metabolic associated fatty liver disease), rats fed a high-fat diet *ad libitum*; TRF (time-restricted feeding), rats fed high-fat diet (60 kcal% fat) strictly between 16:00 and 22:00 every day.

The TICs of different QC samples were overlapped and analyzed. The results showed that the TICs of metabolite detection highly overlapped; the high stability of the instrument guaranteed the repeatability and reliability of the data. PCA showed that the samples demonstrated aggregation within the group and dispersion between groups, with a good sample identification which reflects the results of the subsequent analysis (Figure 6A).

The OPLS-DA models (Figures 6B,C) were qualified (Normal vs. MAFLD, *R*^2^X = 0.731, *R*^2^Y = 0.999, *Q*^2^ = 0.982; MAFLD vs. TRF, *R*^2^X = 0.717, *R*^2^Y = 0.996, *Q*^2^ = 0.843).

**Figure 7:**
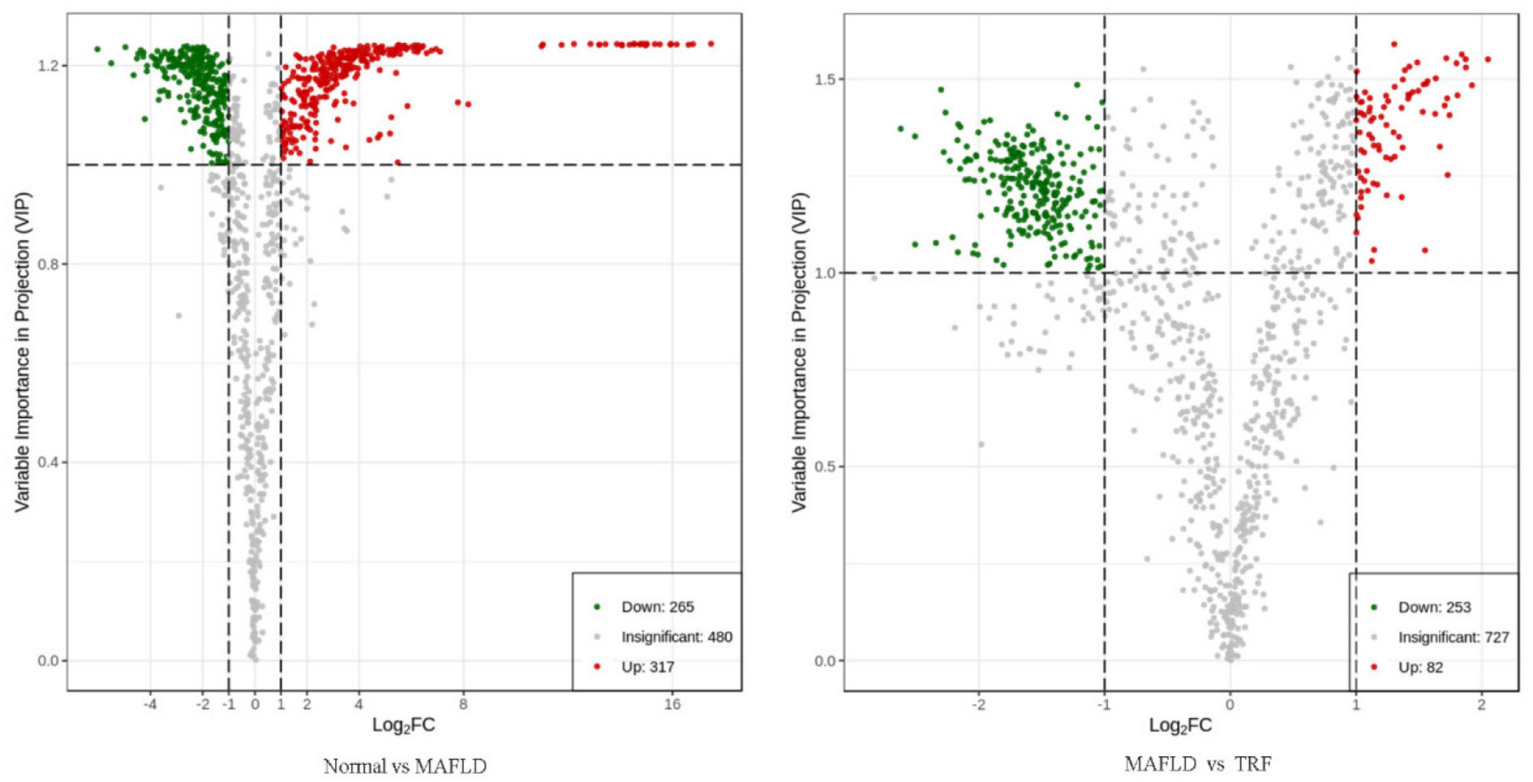
Volcano plot of differential metabolites in liver tissue. Using FC ≥ 2 or ≤ 0.5 and VIP ≥ 1 as criteria, a group of lipids with significant differences were screened. Lipids with significant differences are shown as red (upregulated) or green (downregulated) dots, while gray dots indicate lipids with no significant differences. Normal, rats fed a normal diet *ad libitum*; MAFLD (metabolic associated fatty liver disease), rats fed a high-fat diet *ad libitum*; TRF (time-restricted feeding), rats fed high-fat diet (60 kcal% fat) strictly between 16:00 and 22:00 every day.

**Figure 8:**
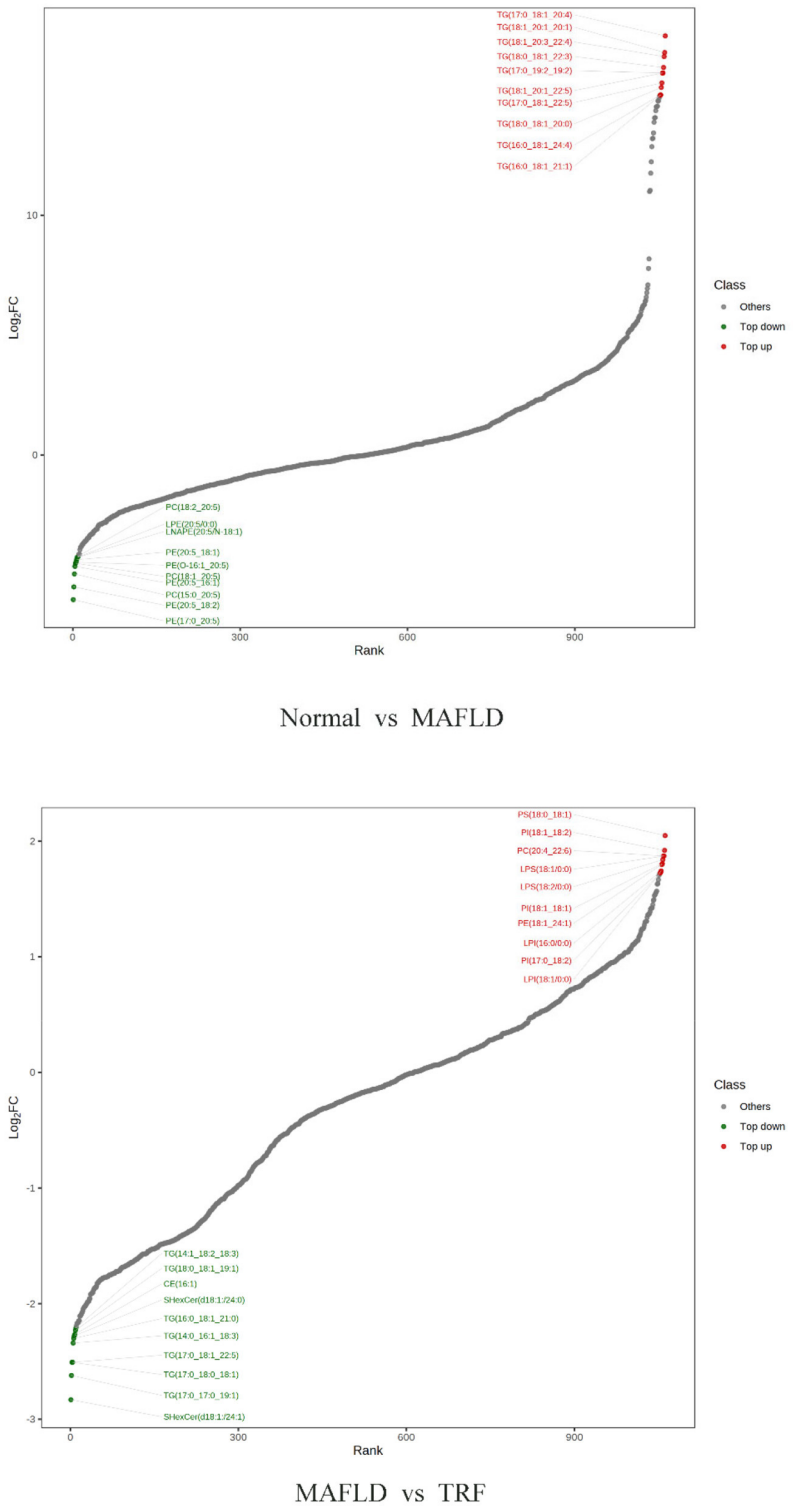
Ten differential metabolites with the most significant upregulation and downregulation in liver tissue. Normal, rats fed a normal diet *ad libitum*; MAFLD (metabolic associated fatty liver disease), rats fed a high-fat diet *ad libitum*; TRF (time-restricted feeding), rats fed high-fat diet (60 kcal% fat) strictly between 16:00 and 22:00 every day.

A total of 1,062 metabolites were detected. Compared with the Normal group, the levels of 317 lipids, including that of TG (17:0_−_18:1_−_20:4) were higher, whereas those of 265 lipids, including phosphatidyl ethanolamine (PE) (17:0_−_20:5) were downregulated in the MAFLD group (*P* < 0.05). Compared with the MAFLD group, the levels of 253 lipids, including that of TG (17:0_−_18:1_−_22:5) were lower, while 82 lipids such as phosphatidylcholine (PC) (20:4_−_22:6) were upregulated in the TRF group (*P* < 0.05; Figures 7, 8). There were 129 identical differential metabolites in the three groups (Figure 6D).

**Figure 9:**
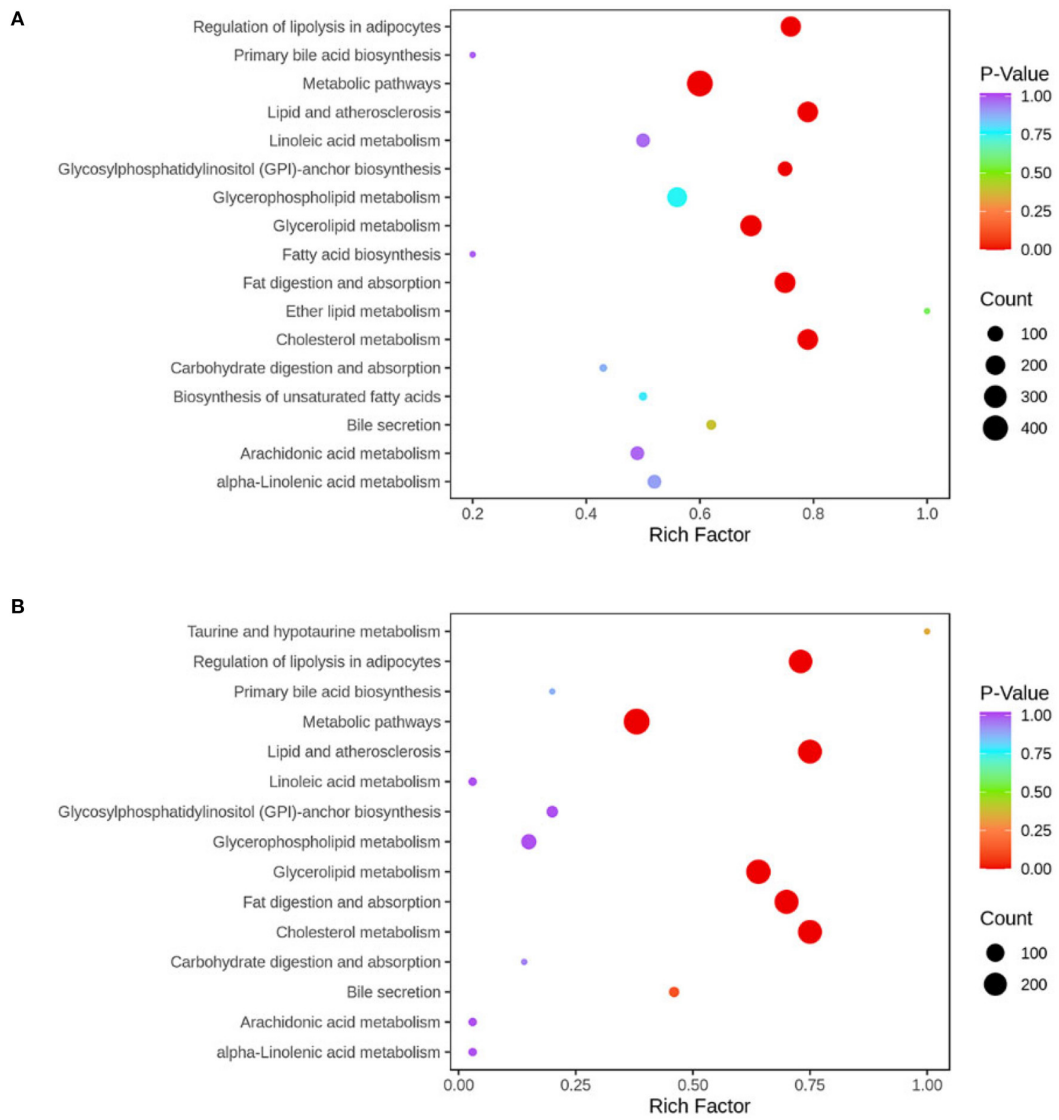
KEGG pathway was enriched according to the results of differential metabolites in liver tissue. The abscissa represents the Rich factor corresponding to each pathway, the ordinate represents the pathway name, and the color of the point indicates the *p*-value: the redder the point, the more significant the enrichment. The size of the dot represents the number of enriched differential metabolites. Normal, rats fed a normal diet *ad libitum*; MAFLD (metabolic associated fatty liver disease), rats fed a high-fat diet *ad libitum*; TRF (time-restricted feeding), rats fed high-fat diet (60 kcal% fat) strictly between 16:00 and 22:00 every day. **(A)** Normal vs. MAFLD; **(B)** MAFLD vs. TRF.

KEGG enrichment analysis of differential metabolites showed that the pathways involved in the observed results mainly included metabolic pathways, fat digestion and absorption, regulation of lipolysis in adipocytes, lipid and atherosclerosis, cholesterol metabolism, and glycerolipid metabolism (Figure 9).

### Expression of genes associated with lipid metabolism in the liver

**Figure 10:**
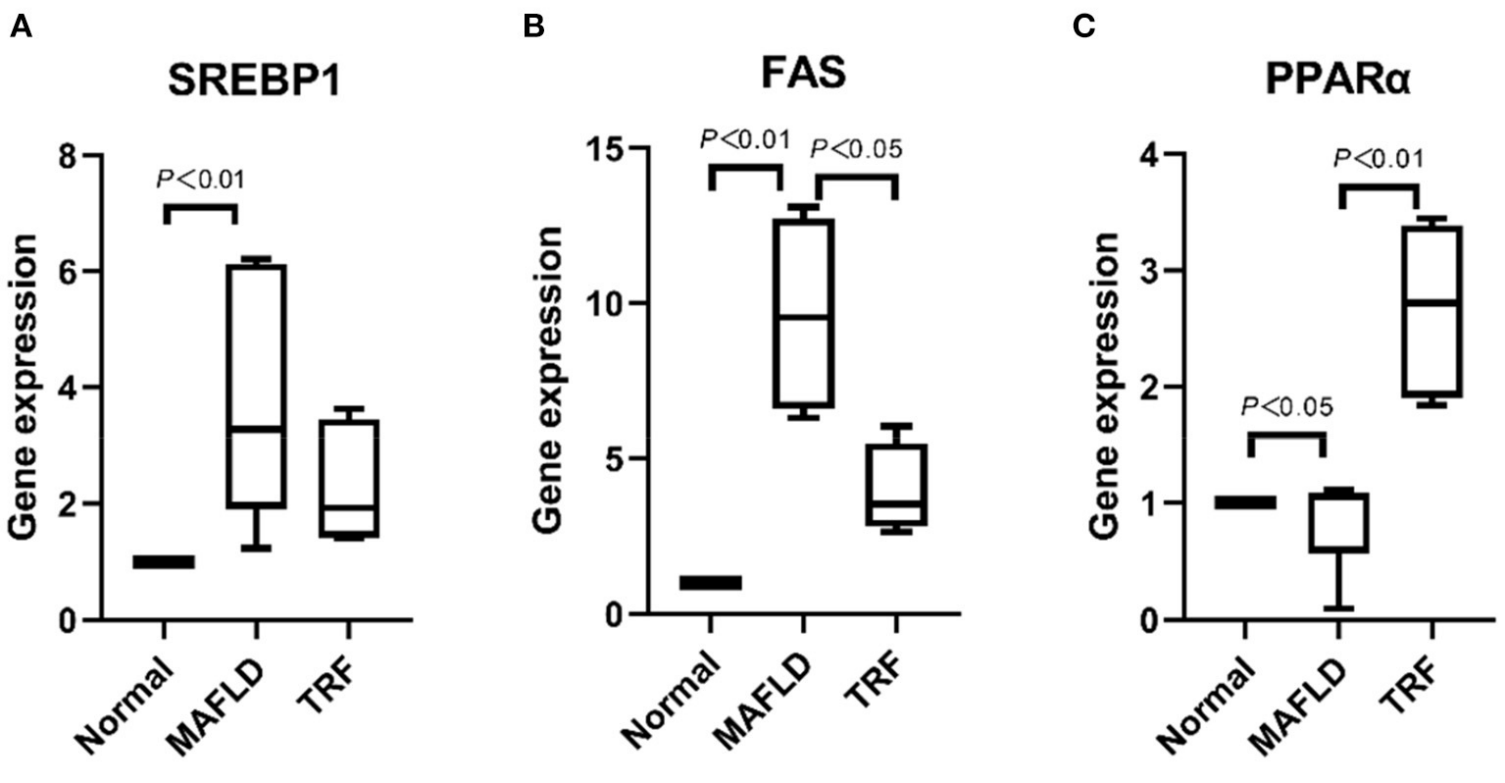
The mRNA levels associated with lipid metabolism in liver. **(A)** SREBP1, **(B)** FAS, and **(C)** PPARα. The data are presented as percentile. Normal, rats fed a normal diet *ad libitum*; MAFLD (metabolic associated fatty liver disease), rats fed a high-fat diet *ad libitum*; TRF (time-restricted feeding), rats fed high-fat diet (60 kcal% Fat) strictly between 16:00 to 22:00 every day.

As shown in Figures 10A,B, the expression levels of lipid synthesis genes *SREBP-1c* and *FAS* in liver tissues were significantly higher in the MAFLD group than in the Normal group (*P* < 0.01), whereas the expression level of *FAS* in the 6 h TRF group was lower than that in the MAFLD group (*P* < 0.05).

Meanwhile, the expression level of the lipid oxidation gene *PPAR*α in the MAFLD group was lower than that in the Normal group (*P* < 0.05). Compared with the MAFLD group, the 6 h TRF group had significantly higher *PPAR*α expression level in the liver tissue (*P* < 0.01; Figure 10C).

## Discussion

In this study, rats adapted to the 6 h TRF mode on day 2, and after 15 weeks of 6 h TRF, the average daily food intake for this group was reduced by 13%, while the body weight, body fat ratio, and liver fat content were lower than those of the MAFLD group. Hundreds of liver lipidomics also improved. Weight loss of 3%–5% within 1 year can improve metabolic syndrome and reverse simple hepatic steatosis, whereas a 7%–10% decrease in body mass can significantly reduce serum amino acid transferase levels and improve non-alcoholic steatohepatitis (6); our results suggest that a 6 h TRF can improve MAFLD.

Unhealthy lifestyles, such as excessive eating and lack of exercise, increase the risk of MAFLD (17). When the body takes in more calories than it burns, the excess calories are stored as fats. Additionally, dietary structure can promote the occurrence of metabolic diseases, such as MAFLD. Excessive intake of fat, cholesterol, and fructose can promote the occurrence of MAFLD; fructose consumption also increases the survival rate of intestinal epithelial cells, which in turn increases the length of intestinal villi, thereby allowing them to absorb more nutrients (18).

Calorie restriction (CR) limits the total number of calories and requires no eating schedule; conversely, IF places more emphasis on restricting the eating schedule, usually for more than 12 h, during which the body will undergo a metabolic switch to burn fat (19) and restore the body's internal clock, which can lead to weight loss and improve metabolic disorders (20–25). Circadian clocks and feeding times regulate the oscillations and levels of hepatic TG (26). Studies have shown that TRF resets the circadian clock in the liver and enhances the transcription of key metabolic regulators of sugar and lipid homeostasis, while dawn-to-sunset fasting is a potentially cost-effective intervention for obesity, metabolic syndrome, and NAFLD (27, 28). TRF can reduce the adverse effects of a high-fat diet by regulating the circadian rhythms of liver lipid metabolism and gut microbiota (29). Indeed, early TRF improves insulin sensitivity, blood pressure, and oxidative stress in men with prediabetes, even without weight loss (30).

The fasting duration of alternate-day fasting is 24 h, which may not be suitable for those with modern lifestyles. Alternatively, if the fasting duration is too short, the effect will be compromised. The 6 h TRF has little effect on work and life, and is similar to the long-standing dictum in China, “no food after noon”, which is conducive to long-term adherence (31).

The results of this study show that compared with the MAFLD group, the 6 h TRF group had significantly higher *PPAR*α mRNA expression in the liver tissue (*P* < 0.01). *PPARs* control various intracellular metabolic processes and contain three subtypes (32), of which *PPAR*α, a member of the nuclear receptor superfamily, is the primary regulator of liver β-oxidation and microsomal ω-oxidation. Moreover, *PPAR*α is involved in mitochondrial fatty acid β-oxidation. Using carnitine palmitoyl transferase-1 as a key enzyme, fatty acids can pass through the mitochondrial inner membrane to the mitochondrial matrix where they are metabolized. The activation of *PPAR*α can reduce the production of TG and fat in the liver and improve MAFLD (33, 34).

Moreover, our results showed that the expression levels of *SREBP-1c* and *FAS* in liver tissues were significantly higher in the MAFLD group than in the Normal group (*P* < 0.01), whereas the expression levels of *FAS* mRNA were lower in the 6 h TRF group than those in the MAFLD group (*P* < 0.05). The *FAS* system is a key multi-enzyme complex in fat synthesis. The downregulation of *FAS* reduces fat synthesis, thus preventing or treating MAFLD (35, 36). *SREBP-1c* plays a major role in the control of lipid production by controlling the expression of several adipogenesis-related genes, and regulates lipid metabolism by promoting lipid synthesis by the liver and inhibiting its transport, while the downregulation of *SREBP-1c* expression restores the balance of liver lipid metabolism to normal (37, 38).

Exercise and hunger can promote *PPAR*α (39), and changes in lipid metabolic pathways during fasting have been reported (9, 19). KEGG enrichment analysis of differential metabolites showed that the pathways involved mainly included glycerolipid metabolism, metabolic pathways, fat digestion and absorption, regulation of lipolysis in adipocytes, lipid, and atherosclerosis. Therefore, we compared the expression of fatty acid metabolism genes to determine the mechanism of 6 h TRF in the treatment of MAFLD. The results showed that, compared with the MAFLD group, the expression of *PPAR*α in the 6 h TRF group was significantly increased, and that of the lipid synthesis gene *FAS* was decreased. Furthermore, by recording the daily food intake, which has not been performed prior to this study, we found that 6 h TRF reduced the daily food intake of rats (16.6 ± 1.6 > 14.4 ± 0.9 g, *P* < 0.001) compared with that of the rats that freely consumed a high-fat diet. This is another significant reason for the decrease in body weight, body fat ratio, and liver fat content in the 6 h TRF group, which is a novel finding of this study (40–42).

The 6 h TRF could reduce the trend of AST, TC, LDL, and UA levels increased with the high-fat diet, however, the difference was not significant. The results also showed that 6 h TRF could increase serum TG level, although not significantly. This suggests that 6 h TRF has no significant effect on the serological indices of MAFLD rats.

Nevertheless, our findings support the hypothesis that 6 h TRF can improve MAFLD; 6 h TRF can not only control the total caloric intake but also reshape metabolic rhythms and regulate the biological clock by restricting eating time. These results provide novel insights pertaining to the prevention and treatment strategies against MAFLD.

## Data availability statement

The raw data supporting the conclusions of this article will be made available by the authors, without undue reservation.

## Ethics statement

The animal study was reviewed and approved by the Biomedical Ethics Committee, School of Medicine, Xi'an Jiaotong University (2021-763).

## Author contributions

JD: conception and design of the study. JD, DF, XJ, SZ, YL, XZ, MLi, MLu, CL, NG, JS, and SD: acquisition of data. JD, DF, and NG: analysis and interpretation of data. JD, JS, and SD: drafting the article. All authors have read and approved the final manuscript.

## Funding

This study was supported by Shannxi Province General Projects-Social Development (2020SF-297), the Fundamental Research Funds for the Central Universities (xjh012019063), and Shaanxi Province Key R&D Program, General Project-Social Development Field (2020SF-180).

## Conflict of interest

The authors declare that the research was conducted in the absence of any commercial or financial relationships that could be construed as a potential conflict of interest.

## Publisher's note

All claims expressed in this article are solely those of the authors and do not necessarily represent those of their affiliated organizations, or those of the publisher, the editors and the reviewers. Any product that may be evaluated in this article, or claim that may be made by its manufacturer, is not guaranteed or endorsed by the publisher.
